# Draft genome sequence of *Magnusiomyces* sp. LA-1 isolated from a C6-C8 acid-producing bioreactor

**DOI:** 10.1128/mra.00935-23

**Published:** 2024-04-29

**Authors:** Kurt Gemeinhardt, Hyojung Park, Jong In Won, Jin Hyung Lee, Ee Taek Hwang, Largus T. Angenent, Byoung Seung Jeon

**Affiliations:** 1 Environmental Biotechnology Group, Department of Geosciences, University of Tübingen, Tübingen, Germany; 2 Center of Convergence Bioceramic Materials, Korea Institute of Ceramic Engineering & Technology, Cheongju-si, Chungcheongbuk-do, South Korea; 3 Department of Food Biotechnology, Dong-A University, Busan, South Korea; 4 AG Angenent, Max Planck Institute for Biology, Tübingen, Germany; 5 Department of Biological and Chemical Engineering, Aarhus University, Aarhus C, Denmark; 6 The Novo Nordisk Foundation CO2 Research Center (CORC), Aarhus University, Aarhus C, Denmark; 7 Cluster of Excellence – Controlling Microbes to Fight Infections, University of Tübingen, Tübingen, Germany; University of Maryland School of Medicine, Baltimore, Maryland, USA

**Keywords:** yeast, lipase, bioreactor, C6 carboxylic acid, C8 carboxylic acid, reactor microbiome, biological chain elongation

## Abstract

Here, we report the draft genome of *Magnusiomyces* sp. LA-1, which was isolated from a C6-C8 carboxylic acid-producing bioreactor. The draft genome of *Magnusiomyces* sp. LA-1 is 19,829,165 bp in length, is divided into six contigs that comprise 6,557 CDS regions, and has a GC content of 34.5%.

## ANNOUNCEMENT

Microbial chain elongation can valorize carbon-rich waste streams into medium-chain carboxylates (C6-C8) (1). We isolated microbial species, which were fed ethanol (300 mM) and acetate (50 mM), from a C6-C8-producing bioreactor (5.0 L volume) in our laboratory. The isolation procedure involved spreading 1 µL of fermentation broth from the bioreactor on LB agar, culturing at 30°C for 2 days, and sequencing the colonies. One isolate showed 99.74% sequence similarity to the *Magnusiomyces capitatus* strain NCCPF (ITS4-ITS5 amplicon), which is a known lipase producer, relevant for biodiesel production (2–4). We designated this strain as *Magnusiomyces* sp. LA-1, from the order Saccharomycetales.

For sequencing, LA-1 was cultivated in liquid LB media at 30°C for 2 days. Genomic DNA was extracted with the YeaStar Genomic DNA Kit (Zymo Research Co. Ltd., USA) and analyzed using the PacBio Sequel System (5) and the Illumina HiSeq X Ten platform (paired-end reads [151 bp]) at Macrogen Inc. (Seoul, South Korea). The library was prepared with the SMRTbell Express Template Prep Kit 2.0. We sheared gDNA with Megaruptor3 (Diagenode). The gDNA size distribution (<17 kb) was estimated with Femto Pulse (Agilent). A 15 kb cutoff was utilized in the BluePippin size selection system. The PacBio Sequel production data consisted of 917,993 subreads and 10,584,985,818 bp. More than 50% of the obtained base reads originated above an N50 read length of 16,998 bp. HGAP Assembly v4.0 (6) was used to assemble subreads generated from the PacBio Sequel system. In the HGAP4 pipeline, PacBio subreads were error corrected with LA4falcon and fc_consensus.py (min. 0.70 identity, min. coverage of 4). Only the longest error-corrected reads (30× coverage, input genome) were used for seed assembly. The TruSeq Nano DNA High Throughput Library Prep Kit (Illumina) facilitated library preparation. Library qualification utilized 4200 TapeStation D1000 screentape (Agilent) and quantification was performed via qPCR (KAPA Library Quantification). We excluded Illumina reads with <90% of bases and a phred quality score of 30 (Q30). The quality-filtered reads were adapter-trimmed using Trimmomatic (v0.38) with the following option: ILLUMINACLIP:Adapter.fasta:2:30:10:8:true LEADING:15 TRAILING:15 SLIDINGWINDOW:4:15 MINLEN:36 (7). After the PacBio subreads were assembled, the obtained Illumina reads were subjected to error correction. The analysis was performed with 8,818,364 filtered Illumina reads (1,330,579,250 bp) from the 14,205,334 raw Illumina reads (2,145,005,434 bp). Pilon (v1.21) (8) was used to revise the assembly. The sites of the protein-encoding genes were predicted after a complete genome analysis, and their functions were annotated. Positions were predicted by Maker (v3.01.03) (9), and Protein BLAST+ (v2.7.1+) was utilized with UniProt Swiss-Prot (v201806)(10). Default parameters were used except where otherwise noted.

As a result of the *de-novo* assembly, six contigs with a total of 1,330,579,250 bp were generated, with an average reference coverage of 444.7×. A total of 6,981 genes were identified after the genes of the assembled genome were annotated, among which 6,557 were predicted to have coding sequences (CDSs); 307 tRNA genes and 118 rRNA genes were identified (Table 1). The average G+C content was 34.5%. BUSCO v3 analysis identified 92.6% of the eukaryote_odb10 BUSCOs in the *Magnusiomyces* sp. LA-1 gene set (11).

**TABLE 1:** Result of assembly of strain LA-1

Contig name	Length	G+C content (%)	No. of CDS	GenBank accession no.
Contig 1	7,365,889	34.5	2,435	CP115648
Contig 2	4,524,598	34.3	1,560	CP115649
Contig 3	3,228,954	34.5	1,070	CP115650
Contig 4	3,088,281	34.9	986	CP115651
Contig 5	1,511,366	35.0	483	CP115652
Contig 6	110,077	24.2	23	CP115653

## Data Availability

This whole-genome project (in GenBank format) and the corresponding raw data files (in FASTQ format) have been deposited under the GenBank accession numbers in Table 1. The BioProject accession number is PRJNA905355, the BioSample accession number is SAMN31871365, and the SRA accession number is SRR23362189. The ITS rRNA gene sequence was submitted to GenBank under accession number OQ755165.

## References

[1] Angenent LT, Richter H, Buckel W, Spirito CM, Steinbusch KJJ, Plugge CM, Strik D, Grootscholten TIM, Buisman CJN, Hamelers HVM. 2016. Chain elongation with reactor microbiomes: open-culture biotechnology to produce biochemicals. Environ Sci Technol 50:2796–2810. doi:10.1021/acs.est.5b04847 26854969

[2] Salgado V, Fonseca C, Lopes da Silva T, Roseiro JC, Eusébio A. 2020. Isolation and identification of Magnusiomyces capitatus as a lipase-producing yeast from olive mill wastewater. Waste Biomass Valor 11:3207–3221. doi:10.1007/s12649-019-00725-7

[3] Subramanya Supram H, Gokhale S, Chakrabarti A, Rudramurthy SM, Gupta S, Honnavar P. 2016. Emergence of Magnusiomyces capitatus infections in Western Nepal . Med. Myco 54:103–110. doi:10.1093/mmy/myv075 26483432

[4] Brejová B, Lichancová H, Brázdovič F, Hegedűsová E, Forgáčová Jakúbková M, Hodorová V, Džugasová V, Baláž A, Zeiselová L, Cillingová A, Neboháčová M, Raclavský V, Tomáška Ľ, Lang BF, Vinař T, Nosek J. 2019. Genome sequence of the opportunistic human pathogen Magnusiomyces capitatus. Curr Genet 65:539–560. doi:10.1007/s00294-018-0904-y 30456648

[5] Kolmogorov M, Yuan J, Lin Y, Pevzner PA. 2019. Assembly of long, error-prone reads using repeat graphs. Nat Biotechnol 37:540–546. doi:10.1038/s41587-019-0072-8 30936562

[6] Chin C-S, Alexander DH, Marks P, Klammer AA, Drake J, Heiner C, Clum A, Copeland A, Huddleston J, Eichler EE, Turner SW, Korlach J. 2013. Nonhybrid, finished microbial genome assemblies from long-read SMRT sequencing data. Nat Methods 10:563–569. doi:10.1038/nmeth.2474 23644548

[7] Bolger AM, Lohse M, Usadel B. 2014. Trimmomatic: a flexible trimmer for illumina sequence data. Bioinformatics 30:2114–2120. doi:10.1093/bioinformatics/btu170 24695404 PMC4103590

[8] Walker BJ, Abeel T, Shea T, Priest M, Abouelliel A, Sakthikumar S, Cuomo CA, Zeng Q, Wortman J, Young SK, Earl AM. 2014. Pilon: an integrated tool for comprehensive microbial variant detection and genome assembly improvement. PLoS One 9:e112963. doi:10.1371/journal.pone.0112963 25409509 PMC4237348

[9] Holt C, Yandell M. 2011. Maker2: an annotation pipeline and genome-database management tool for second-generation genome projects. BMC Bioinformatics 12:1–14. doi:10.1186/1471-2105-12-491 22192575 PMC3280279

[10] Boutet E, Lieberherr D, Tognolli M, Schneider M, Bansal P, Bridge AJ, Poux S, Bougueleret L, Xenarios I. 2016. Uniprotkb/Swiss-PROT, the manually annotated section of the uniprot knowledgebase: how to use the entry view. Methods Mol Biol 1374:23–54. doi:10.1007/978-1-4939-3167-5_2 26519399

[11] Simão FA, Waterhouse RM, Ioannidis P, Kriventseva EV, Zdobnov EM. 2015. BUSCO: assessing genome assembly and annotation completeness with single-copy orthologs. Bioinformatics 31:3210–3212. doi:10.1093/bioinformatics/btv351 26059717

